# Predicting Fusarium Head Blight Resistance for Advanced Trials in a Soft Red Winter Wheat Breeding Program With Genomic Selection

**DOI:** 10.3389/fpls.2021.715314

**Published:** 2021-10-22

**Authors:** Dylan L. Larkin, Richard Esten Mason, David E. Moon, Amanda L. Holder, Brian P. Ward, Gina Brown-Guedira

**Affiliations:** ^1^Department of Crop, Soil, and Environmental Sciences, University of Arkansas, Fayetteville, AR, United States; ^2^USDA-ARS SEA, Plant Science Research, Raleigh, NC, United States; ^3^Department of Crop and Soil Sciences, North Carolina State University, Raleigh, NC, United States

**Keywords:** genomic selection, Fusarium head blight, wheat, resistance, multi-trait genomic selection, forward prediction

## Abstract

Many studies have evaluated the effectiveness of genomic selection (GS) using cross-validation within training populations; however, few have looked at its performance for forward prediction within a breeding program. The objectives for this study were to compare the performance of naïve GS (NGS) models without covariates and multi-trait GS (MTGS) models by predicting two years of F_4:__7_ advanced breeding lines for three Fusarium head blight (FHB) resistance traits, deoxynivalenol (DON) accumulation, Fusarium damaged kernels (FDK), and severity (SEV) in soft red winter wheat and comparing predictions with phenotypic performance over two years of selection based on selection accuracy and response to selection. On average, for DON, the NGS model correctly selected 69.2% of elite genotypes, while the MTGS model correctly selected 70.1% of elite genotypes compared with 33.0% based on phenotypic selection from the advanced generation. During the 2018 breeding cycle, GS models had the greatest response to selection for DON, FDK, and SEV compared with phenotypic selection. The MTGS model performed better than NGS during the 2019 breeding cycle for all three traits, whereas NGS outperformed MTGS during the 2018 breeding cycle for all traits except for SEV. Overall, GS models were comparable, if not better than phenotypic selection for FHB resistance traits. This is particularly helpful when adverse environmental conditions prohibit accurate phenotyping. This study also shows that MTGS models can be effective for forward prediction when there are strong correlations between traits of interest and covariates in both training and validation populations.

## Introduction

Resistance to the disease Fusarium head blight (FHB) is important in wheat (*Triticum aestivum* L.) production, particularly in the Southeastern US. Fusarium head blight is a fungal disease caused by *Fusarium graminearum* and incurs nearly US$4.2 billion in losses annually (Wilson et al., 2017). The *F. graminearum* pathogen produces the mycotoxin deoxynivalenol (DON), which is harmful for humans and animals that consume infected grain (FDA, 2010; Sobrova et al., 2010).

Traditionally, wheat breeders have primarily relied on phenotypic selection within their breeding programs to advance breeding material. However, phenotypic selection has its limitations, especially with low-heritability traits of interest that are difficult to phenotype. Difficulties with phenotyping are also compounded by genotype × environment interactions that can lead to differential responses between genotypes across environments, reducing the accuracy of selections. Alternatives to phenotypic selection include marker assisted selection (MAS) and genomic selection (GS). Marker assisted selection can be effective for qualitative traits controlled by one or two genes or quantitative traits that are controlled by large-effect quantitative trait loci (QTL) (Xu and Crouch, 2008). However, MAS is less effective for complex quantitative traits controlled by many small-effect QTL (Bernardo and Yu, 2007; Heffner et al., 2009). Genomic selection is an effective alternative to both phenotypic selection and MAS, in that it incorporates allelic effects across the entire genome, making it ideal for quantitative traits. Genomic selection can also reduce the time within a breeding cycle, as two rounds of GS can be performed compared to one cycle of phenotypic selection allowing for greater genetic gain over time (Bernardo and Yu, 2007; Heffner et al., 2009; Asoro et al., 2013; Rutkoski et al., 2015).

Genomic selection was first applied to animal breeding, particularly in the dairy industry, but it has since been adapted by plant breeders over the last decade (Meuwissen et al., 2001; Heffner et al., 2009). Genomic selection uses a training population (TP), a panel of lines that have been phenotyped for a trait of interest and genotyped using whole-genome sequencing, to train a genomic prediction model. The genomic prediction model then uses relatedness between all genotypes to obtain genome-estimated breeding values (GEBVs) for breeding lines, otherwise known as the validation population (VP), that have only been genotyped. The breeder can then make selections based on the GEBVs for a trait of interest (Meuwissen et al., 2001).

Most studies involving GS have focused on increasing prediction accuracy by manipulating the TP and subsequently evaluating model performance through cross-validation within the TP (Habier et al., 2007; Heffner et al., 2009; Jannink et al., 2010; Combs and Bernardo, 2013; Akdemir et al., 2015; Isidro et al., 2015; Larkin et al., 2019). Many have also investigated the genomic prediction model used for GS analysis (Heslot et al., 2012). While these methods are valuable, few have researched the effectiveness of applying GS in breeding programs for forward prediction of breeding lines (Bernardo, 2016). However, when investigated, many have seen mixed results regarding prediction accuracy of forward prediction, compared to cross-validated prediction accuracy within TPs (Asoro et al., 2013; Combs and Bernardo, 2013; Massman et al., 2013; Michel et al., 2017; Belamkar et al., 2018; Calvert et al., 2020). Additionally, there are few, if any, studies that focus on forward prediction for FHB resistance in wheat as opposed to grain yield (GY) (Michel et al., 2016, 2017; Belamkar et al., 2018; Calvert et al., 2020).

In an evaluation of GS in the Kansas State University wheat breeding program, GS was used to predict GY in a TP where the prediction accuracy was between *r* = 0.31 and *r* = 0.47. However, when the TP was used for forward prediction, the highest prediction accuracy between the GEBVs for GY in the preliminary yield trials (PYTs) and the actual phenotypic results for GY was *r* = −0.16 (Calvert et al., 2020). This trend was also observed in an evaluation of the University of Nebraska wheat breeding program, where GY data from PYTs from three years were used to predict the performance of a fourth year. When no lines for the fourth year were included in the TP, prediction accuracies for GY were between *r* = 0.22 and *r* = 0.26. However, as more lines from the fourth year were included in the TP, the prediction accuracy of GY for the remaining lines in the fourth year increased to between *r* = 0.37 and *r* = 0.52, when 90% of the lines from the fourth year were included in the TP (Belamkar et al., 2018). Phenotypic selection and GS were also compared in terms of selection accuracy between the PYT and advanced yield trial generations. Genomic selection outperformed phenotypic selection during the 2012 and 2015 seasons, where Nebraska experienced severe drought and disease stress. Even still, prediction accuracies were low, indicating that prediction accuracy is not the best indicator of GS success for forward prediction (Belamkar et al., 2018). Another study using forward prediction for GY in wheat adapted to central Europe found that the use of GS (*r* = 0.39) to select high performing lines for multiple-environment trials was far better than phenotypic selection (*r* = 0.21) (Michel et al., 2017).

In addition to traditional GS, researchers have begun investigating the efficacy of multi-trait GS (MTGS). Multi-trait GS uses mixed models that incorporate secondary traits that are genetically correlated with a trait of interest as covariates to improve the prediction accuracy for the trait of interest (Calus and Veerkamp, 2011; Jia and Jannink, 2012; Covarrubias-Pazaran et al., 2018). Multi-trait GS can improve prediction accuracies for low-heritability traits when high-heritability secondary traits are used as covariates (Calus and Veerkamp, 2011; Guo et al., 2014; Jia et al., 2018). Many studies have evaluated MTGS models for cross-validation, particularly for GY in wheat using high-throughput phenotyping traits (Rutkoski et al., 2016; Sun et al., 2017; Crain et al., 2018; Lozada and Carter, 2019; Guo et al., 2020). Others have evaluated resistance traits related to FHB in wheat using phenological traits, such as heading date (HD) and plant height (PH), or other FHB resistance traits as covariates (Rutkoski et al., 2012; Schulthess et al., 2018; Steiner et al., 2019; Larkin et al., 2020; Moreno-Amores et al., 2020). Few have evaluated the use of MTGS for forward prediction. One study used high-throughput phenotyping traits as a covariate in a MTGS model for forward prediction of GY in wheat, though the prediction accuracy was unfavorable unless a large TP was used (Calvert et al., 2020). Therefore, our aim is to validate the use of MTGS models compared to naïve GS (NGS) models to predict FHB resistance in wheat, using secondary FHB resistance traits regularly collected throughout the season within a breeding program based on results from Larkin et al. (2020).

The University of Arkansas soft red winter wheat (SRWW) breeding program makes over 800 unique crosses per year. Progenies are then tested over the following 10 seasons prior to releasing a new cultivar (Mason et al., 2018). Breeding lines are not evaluated for FHB resistance traits until the F_4:7_ advanced (ADV) and F_4:8_ elite (ARE) trials, where they are evaluated in misted and inoculated FHB disease nurseries at two locations in a RCBD design with two replications. Selections are made based on three FHB resistance traits: type II resistance, which is resistance to the spread of FHB within a spike, otherwise known as severity (SEV) (Schroeder and Christensen, 1963); type III resistance, or resistance to Fusarium damaged kernels (FDK) (Argyris et al., 2003; Goral et al., 2019); and type IV resistance, or resistance to DON accumulation (Mesterhazy, 1995).

Some elite lines are also grown in regional statewide variety testing trials, as well as the USDA-ARS Uniform Eastern (UE) and Southern nurseries (US), Southeastern University Grains (Sungrains) cooperative nurseries, and foundation seed increases. The UE and US nurseries include approximately 36 elite breeding lines from public and private SRWW breeding programs in the Southern and Eastern US, grown between 22 and 36 locations with between one and three replications per location annually (Boyles et al., 2019). The Sungrains cooperative consists of Southeastern US SRWW breeding programs that performs regional testing within the Southeastern US (Harrison et al., 2017; Johnson et al., 2017; Mason et al., 2018; Boyles et al., 2019). Select breeding lines from the ADV and ARE are grown in these regional Sungrains nurseries.

In theory, GS can improve selection accuracy in the early generations of the breeding program for FHB resistance traits while also reducing time and resources spent for phenotyping. In this study, we evaluated the selection accuracy of GS from the advanced through elite generations and compared to phenotypic selection through forward prediction using NGS and MTGS models. The three goals for this study were to: (1) compare NGS and MTGS with phenotypic selection for three FHB resistance traits, including DON, FDK, and SEV for new breeding lines that have not been phenotyped at the advanced generations; (2) compare the selection accuracy between NGS, MTGS, and phenotypic selection between the advanced and elite generations of the University of Arkansas SRWW breeding program; and (3) compare the response to selection between NGS, MTGS, and phenotypic selection between the advanced and elite generations of the University of Arkansas SRWW breeding program.

## Materials and Methods

### Plant Materials

#### Breeding Materials

Two generations of the ADV trials, 2017–2018 and 2018–2019, consisting of F_4:7_ breeding lines from the University of Arkansas wheat breeding program and doubled haploid (DH) lines developed through the Sungrains cooperative, were used as VPs to predict three FHB traits, DON, FDK, and SEV. Approximately 20% of breeding lines from the ADV18 and ADV19 yield trials were selected and advanced to the ARE19 and ARE20 yield trials for the 2018–2019 and 2019–2020 growing seasons, respectively. Genotypes were advanced based on both GS and phenotypic selection (Table 1).

**TABLE 1:** Description of the number of genotypes, composition, and experimental design of two generations of F_4:7_ advanced nurseries (ADV), and F_4:8_ elite nurseries (ARE), as well as the initial training population (TP18_FHB) used to predict three Fusarium head blight (FHB) resistance traits, including deoxynivalenol (DON) accumulation, Fusarium damaged kernels (FDK), and severity (SEV).

Trial^a^	Generation^b^	Conventional lines	DH lines	Total	Location(s)	Rep(s)	Design^c^
TP18_FHB	–	355	–	355	9	2	RCBD
ADV18	F4:7/DH	64	40	104	2	2	RCBD
ADV19	F4:7/DH	50	70	120	2	2	RCBD
ARE19	F4:8/DH	16	6	22	2	2	RCBD
ARE20	F4:8/DH	12	11	23	1	2	RCBD

*^a^Trial types and the years each were grown. TP18_FHB was grown over four years between 2013–2014 and 2016–2017; 18, 2017–2018; 19, 2018–2019; 20, 2019–2020.*

*^b^Breeding trials consisted of conventionally bred genotypes as well as doubled haploid (DH) genotypes.*

*^c^RCBD, randomized complete block design.*

#### Training Populations

A population of 355 SRWW genotypes was used as the initial 2018 TP (TP18_FHB) for this study to predict GEBVs for DON, FDK, and SEV in the ADV18 trial. The population consisted of 187 genotypes from the University of Arkansas, 87 from Louisiana State University, 40 from North Carolina State University, 38 from the University of Georgia, and one genotype each from Syngenta AG, Pioneer Hi-Bred International, Inc., and Virginia Polytechnic Institute and State University (Larkin et al., 2020). The 2019 TP (TP19_FHB) for the three FHB traits consisted of the 355 genotypes from TP18_FHB, as well as the 104 genotypes from the ADV18 trial.

### Experimental Design and Trait Measurements

Winter wheat is planted during the fall and harvested during the late spring in the southern United States, therefore the growing season spans two years. The TP18_FHB genotypes were evaluated for three FHB resistance traits, including DON, FDK, and SEV, over four seasons between 2014 and 2017 at two locations, at the Milo J. Shult Agricultural Research and Extension Center in Fayetteville, AR, United States (FAY) and the Newport Research and Extension Center near Newport, AR, USA (NPT). The data collection and experimental design methods were outlined in Larkin et al. (2020), as TP18_FHB was the same population used in their study.

The AVD18, ADV19, and ARE19 FHB nurseries for the 2017–2018 and 2018–2019 growing seasons were grown at two locations, FAY and NPT, in a randomized complete block design (RCBD) with two replications per location using the same methods described with respect to the TP18_FHB and TP19_FHB populations in Larkin et al. (2020). This was also the case for the ARE20 FHB nursery; however, it was only grown in NPT during the 2019–2020 season due to poor growing conditions in FAY. Data were also collected for HD, PH, DON, FDK, and SEV for the FHB nurseries using methods described in Larkin et al. (2020).

### Phenotypic Data Analyses

Phenotypic data was analyzed using a single stage mixed linear model within the PROC MIXED procedure in SAS 9.4 to obtain adjusted means for HD, PH, DON, FDK, and SEV (SAS Institute Inc., Cary, NC, United States). The following model was fit to the phenotypic data:


$$y_{ijk}=\mu +genotype_{i}+rep{\left(env\right)}_{jk}+env_{k}+{\left(genotype\times env\right)}_{ik}+\varepsilon_{ijk}$$

where *y*_*ijk*_ is the observed phenotype, μ is the population mean, *genotype*_*i*_ is the fixed effect of the *i*^*th*^ genotype, *rep(env)_*jk*_* is the random effect of the *j*th replication nested within the *k*th location-year (or location) (*env*), *env*_*k*_ is the random effect of the *k*th location-year (or location), (*genotype* × *env*)*_*ik*_* is the random effect of the interaction between genotype and location-year (or location), and ε_*ijk*_ is the residual error term, where ε_*ijk*_ ∼ *N*(0,***I***σ^2^_ε_), where ***I*** is an identity matrix and σ^2^_ε_ is the residual error variance.

Phenotypic Pearson correlations were calculated between DON, FDK, HD, PH, and SEV within TP18_FHB and TP19_FHB as well as the ADV and ARE FHB nurseries using the multivariate function in JMP Pro 15.2.0 software (SAS Institute Inc., Cary, NC). Entry mean-based broad-sense heritability (*H*^2^) was calculated for each trait using the following equation:


$$H^{2}=\frac{\sigma_{genotype}^{2}}{\sigma_{genotype}^{2}+\frac{\sigma_{genotype\times env}^{2}}{n_{env}}+\frac{\sigma_{\varepsilon }^{2}}{n_{env}\times n_{rep}}}$$

where σ^2^_*genotype*_ is the genotypic variance, σ^2^_*genotype* × *env*_ is the variance of the interaction between genotype and location-year, *n*_*env*_ is the number of location-years where the trait was evaluated, σ^2^_ε_ is the residual error variance, and *n*_*rep*_ is the number of replications within each location-year. Variance components were obtained from the single stage mixed linear model described above for each trait using the PROC MIXED procedure in SAS 9.4. Narrow-sense heritability (*h*^2^) was calculated using the “marker_h^2^” function within the “heritability” package in R v4.0.3 software for TP19_FHB due to a lack of shared genotypes within the TP (Kruijer et al., 2015; R Core Team, 2020). The analysis used a genome relationship matrix obtained from the “A.mat” function within the “rrBLUP” package in Rv4.0.3 software using the marker set described below as well as the abovementioned phenotypic data (Endelman, 2011; Endelman and Jannink, 2012; R Core Team, 2020).

### Genotyping by Sequencing

All genotypes were genotyped using genotyping by sequencing (GBS) using methods described in Larkin et al. (2020). Single nucleotide polymorphism (SNP) calling was performed using the TASSEL 5.0 GBSv2 pipeline using 64 base tag length and a minimum tag count of five (Bradbury et al., 2007). Reads were aligned to the International Wheat Genome Sequencing Consortium (IWGSC) RefSeq v1.0 “Chinese Spring” wheat reference sequence (Appels et al., 2018) using the Burrows-Wheeler aligner version 0.7.17 (Li and Durbin, 2009).

Raw SNP data generated from the TASSEL pipeline were filtered using PLINK software (Purcell et al., 2007) to remove taxa with more than 85% missing data and heterozygosity greater than 30%. Genotypic data were then filtered to select for biallelic SNPs with minor allelic frequency of greater than five percent, less than 20% missing data, and heterozygosity less than or equal to 10%. Missing marker data were then imputed using BEAGLE software, based on windows encompassing the entire chromosome (Browning et al., 2018). Markers were again filtered after imputation to select SNP markers with minor allele frequency greater than five percent and heterozygosity of less than equal to 10% using PLINK software. Markers aligning to unassembled contigs were also removed for a final genotypic dataset of 5,202 SNP markers.

Principal component analyses were performed within each of the TPs to evaluate the genetic relationships between subpopulations using the PCA function in TASSEL 5.0. These relationships between the first three principal components were visualized for each TP using the “scatterplot3d” package in R v4.0.3 software (Ligges et al., 2018; R Core Team, 2020).

### Genomic Selection

Two different models were tested for both TPs to obtain GEBVs for DON, FDK, and SEV for the ADV18 and ADV19 trials. The first model was a naïve genomic BLUP (GBLUP) model with no covariates (NGS). The second model was a MTGS GBLUP model where DON was predicted using FDK and HD as covariates, FDK was predicted using DON and SEV as covariates, and SEV was predicted using FDK and PH as covariates. The optimal covariate combinations for the MTGS models were determined in Larkin et al. (2020) for the FHB traits.

#### Cross Validation

Mean prediction accuracies between the NGS and MTGS models for each TP were obtained using a five-fold cross-validation analysis performed using the Genomic Selection function in TASSEL 5.0 (Bradbury et al., 2007). The GBLUP model used for the analyses is described as follows:


$$y=\text{X}\beta +\text{Z}u+\varepsilon_{i}$$

where *u* is a vector of genotype effects, which is assumed to have a normal distribution *u*∼$N\left(0,\text{G}\sigma_{u}^{2}\right)$, where ***G*** is the genomic relationship matrix, obtained using the Kinship function within TASSEL 5.0, which uses the same methodology as the “rrBLUP” package in R (Endelman, 2011; Endelman and Jannink, 2012), and $\sigma_{u}^{2}$ is the variance of the individual genotype effects; β is a vector of fixed effects; ***X*** is a design matrix relating fixed effects to phenotypic observations (*y*); ***Z*** is a design matrix relating random effects to phenotypic observations; and ε*_*i*_* is the residual error at the *i*th locus, which is assumed to have a normal distribution $\varepsilon_{i}∼N\left(0,\text{I}\sigma_{\varepsilon }^{2}\right)$, where ***I*** is the identity matrix and σ^2^_ε_ is the residual error variance. The GEBV from the GBLUP model is equivalent to the sum of all allele effects of a genotype from the ridge regression BLUP (RR-BLUP) model (VanRaden, 2008; Endelman, 2011).

The five-fold cross-validation approach randomly divided the TP into five equal sized groups. Four of the five groups were then used as the TP to train the GBLUP model to calculate GEBVs for the fifth group, serving as the VP, where the phenotypic values were set as missing. In the case of the MTGS models, the phenotypic data for the covariate traits were used as a fixed effect in the model. The GEBVs calculated for the VP were compared to the actual phenotypic values using a Pearson correlation. The five-fold cross-validation process was repeated over 100 iterations for a total of 500 iterations. The mean prediction accuracies between the NGS and MTGS models were compared between both TPs using a generalized linear mixed model (GLMM) and Fisher’s LSD with an α of 0.05, implemented in PROC GLIMMIX in SAS 9.4. Mean prediction accuracy comparisons between the NGS and MTGS models for each TP were visualized using the “yarrr” package in R v4.0.3 (Phillips, 2017; R Core Team, 2020).

#### Forward Prediction

Both TPs were then used to obtain predictions for their respective VPs using the NGS and MTGS GBLUP models associated with each trait. For example, TP18_FHB was used to calculate GEBVs for DON, FDK, and SEV for the ADV18 trial using the NGS and MTGS models (Table 2).

**TABLE 2:** Descriptive statistics, Pearson phenotypic correlations, and heritabilities (*H*^2^) for adjusted means for two training populations, two advanced F_4:7_ nurseries, and two elite F_4:8_ nurseries for three Fusarium head blight (FHB) resistance traits, including deoxynivalenol (DON), Fusarium damaged kernels (FDK), and severity (SEV) as well as heading date (HD) and plant height (PH).

Trial^a^	Trait	Summary statistics						Correlations			
		Mean	Min	Max	Range	SD	*H* ^2^ ^b^	DON^c^	FDK	SEV	HD^d^
FHB_TP18	DON	10.53	0.08	92.80	92.72	11.35	0.74	−	−	−	−
	FDK	32.49	0.00	100.00	100.00	29.93	0.79	0.40***	−	−	−
	SEV	27.88	0.00	100.00	100.00	25.78	0.82	0.32***	0.73***	−	−
	HD	94.69	74.00	118.00	44.00	10.19	0.90	0.25***	−0.05^ns†^	−0.10*	−
	PH^e^	90.27	56.46	121.87	65.40	10.06	0.91	0.01^ns^	−0.29***	−0.36***	0.34***
FHB_TP19	DON	14.26	6.15	37.50	31.35	4.59	0.60	−	−	−	−
	FDK	38.22	6.00	92.12	86.12	14.86	0.68	0.45***	−	−	−
	SEV	28.67	3.75	91.71	87.96	12.97	0.93	0.12*	0.55***	−	−
	HD	97.91	86.76	116.50	29.74	8.30	0.92	0.31***	0.02^ns^	−0.54***	−
	PH	90.40	71.12	113.03	41.91	6.96	0.74	0.00^ns^	−0.29***	−0.31***	0.16***
ADV18	DON	16.64	3.60	51.50	47.90	7.60	0.62	−	−	−	−
	FDK	39.32	2.00	75.00	73.00	16.29	0.77	0.62***	−	−	−
	SEV	15.44	0.00	85.00	85.00	14.88	0.38	0.27**	0.54***	−	−
	HD	112.22	108.00	117.00	9.00	2.16	0.90	0.28**	−0.10^ns^	−0.34***	−
	PH	89.46	68.58	119.38	50.80	8.53	0.71	−0.05^ns^	−0.22*	−0.18*	0.25***
ADV19	DON	10.09	0.12	74.50	74.38	10.08	0.61	−	−	−	−
	FDK	31.01	0.00	98.00	98.00	23.94	0.83	0.86***	−	−	−
	SEV	25.60	0.00	95.00	95.00	25.54	0.45	0.76***	0.86***	−	−
	HD	102.38	97.00	109.00	12.00	2.35	0.81	0.10^ns^	−0.04^ns^	−0.17^ns^	−
	PH	81.20	63.50	101.60	38.10	7.16	0.74	0.29***	0.09^ns^	0.01^ns^	0.35***
ARE19	DON	8.51	0.59	64.10	63.51	8.32	0.50	−	−	−	−
	FDK	27.04	1.00	95.00	94.00	21.06	0.71	0.84***	−	−	−
	SEV	23.55	0.00	90.00	90.00	22.87	0.43	0.74***	0.86***	−	−
	HD	102.20	98.00	108.00	10.00	2.27	0.84	0.01^ns^	−0.28^ns^	−0.28^ns^	−
	PH	80.06	53.34	93.98	40.64	7.14	0.76	0.21^ns^	−0.12^ns^	−0.18^ns^	0.41*
ARE20	DON	7.30	0.99	19.30	18.31	3.95	0.78	−	−	−	−
	FDK	15.21	2.00	60.00	58.00	12.49	0.84	0.78***	−	−	−
	SEV	16.83	0.00	60.00	60.00	13.11	0.76	0.65***	0.82***	−	−
	HD	99.49	94.00	111.00	17.00	3.39	0.76	0.09^ns†^	0.09^ns^	−0.08^ns^	−
	PH	89.69	76.20	101.60	25.40	6.20	0.87	0.01^ns^	−0.05^ns^	−0.11^ns^	0.48**

*^a^TP, training population; ADV, F_4:7_ advanced FHB trial; ARE, F_4:8_ elite FHB trial.*

*^b^Broad-sense heritability for FHB_TP18, ADV18, ADV19, ARE19, and ARE20 calculated using entry-mean based heritability. Narrow-sense heritability was calculated for FHB_TP19.*

*^c^DON was recorded in μg g^–1^, whereas FDK and SEV were recorded in percentage.*

*^d^Heading date was recorded as day of year after 1st of January, when 50% of the heads were emerged from the flag leaf.*

*^e^Plant height was recorded in inches from the surface of the soil to the tip of the head minus awns if present, but reported in centimeters here.*

*Significant at the 0.05 probability level.

**Significant at the 0.01 probability level.

***Significant at the 0.001 probability level.

*^†^ns, nonsignificant at the 0.05 probability level.*

Once GEBVs for each trait for each model were obtained, GEBVs were compared to the adjusted mean of the trait of interest for each genotype in the following generation using a Pearson correlation using the multivariate function in JMP 15.2.0 software. For example, GEBVs for DON obtained for ADV18 were compared to the adjusted mean DON for each genotype across the ADV18 and ARE19 generations. This serves as a form of prediction accuracy for the respective model and TP. A scatterplot visualizing the comparison between GEBVs and adjusted means across years for each genotype, as well as individual genotypes advanced to the next generation, was created using the “ggplot2” package in R v4.0.3 for each model for each TP (Wickham et al., 2016; R Core Team, 2020). Selection accuracy was also determined as the percentage of genotypes advanced to the ARE generation that were above average based on GEBVs from the NGS or MTGS models as well as above average based on phenotypic values.

Response to selection was also compared between the NGS and MTGS models and phenotypic selection, based on the adjusted means from the ADV generations for FHB traits, using a selection pressure of 50%. The response to selection formula is as follows:


$$R=H^{2}S$$

where *H*^2^ was the broad-sense heritability calculated as above, and *S* is the selection differential, calculated as *S* = μ_*Selected*_−μ_*Unselected*_ where μ_*Selected*_ is the mean of the phenotypic data for the top 50% of genotypes selected for genotypes in the ARE generations using either phenotypic selection, NGS, or MTGS, and μ_*Unselected*_ is the mean of the full unselected population of the genotypes in the ARE generation of the breeding cycle (Falconer and McKay, 1996; Arruda et al., 2016b; Lozada et al., 2020).

## Results

### Variation in Fusarium Head Blight Resistance Traits

Both FHB TPs as well as the ADV and ARE FHB trials had significant variation for all five traits. The ADV18 FHB trial had the highest mean DON and FDK, but it also had the lowest mean SEV. The ARE20 FHB trial had the lowest mean DON and FDK, likely due to stronger genetic resistance (Table 2). All trials also had significant correlations between the three FHB traits. Correlations between DON and HD were consistently positive, however, the correlations were not significant with smaller population sizes, while DON was significantly correlated with PH only in ADV19. There were generally negative correlations between FDK and PH apart from ADV19, however, the significance of the correlations between FDK and PH were not significant with smaller population sizes. There were strong negative correlations between SEV and HD and PH for nearly all trials, however, they were not significant for smaller populations. High heritability was also observed for all three FHB traits in addition to HD and PH (Table 2).

### Population Structure

Genotyping by sequencing identified 5,202 SNPs across the entire wheat genome after filtering and imputation. The number of SNP markers were unevenly distributed between genomes, where the B genome had the largest number of markers (2,315), followed by the A (2,210) and D (677) genomes, which was consistent with other studies using GBS SNPs (Arruda et al., 2016a; Larkin et al., 2020). The chromosome with the largest number of SNPs was 3B at 477, while the chromosome with the smallest number was 4D (38). The proportion of heterozygosity within the dataset was 2.5% and the average minor allele frequency was 21.6%.

**FIGURE 1:**
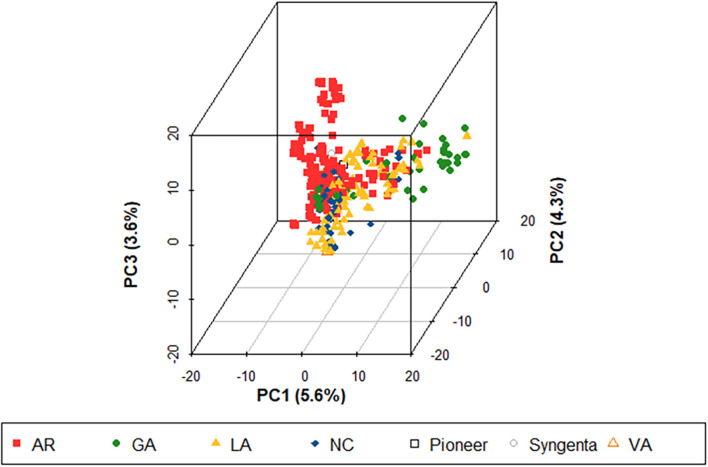
Population structure of 355 soft red winter wheat genotypes using 5,202 single nucleotide polymorphism (SNP) markers. This population represents the training population used to predict three Fusarium head blight (FHB) resistance traits including deoxynivalenol (DON) concentration, Fusarium damaged kernels (FDK), and severity (SEV) (TP18_FHB) for the 2018 advanced Fusarium head blight trial (ADV18). Colors represent the origin of the genotypes. AR, developed at the University of Arkansas, Fayetteville; GA, developed at the University of Georgia, Athens; LA, developed at Louisiana State University, Baton Rouge; NC, developed at North Carolina State University, Raleigh; Pioneer, developed by Pioneer Hi-Bred International; Syngenta, developed by Syngenta and AgriPro; and VA, developed by Virginia Polytechnic Institute and State University, Blacksburg; PC, principal component.

The PCA of the initial TP18_FHB population showed two primary clusters within the population. Genotypes from all breeding programs appeared in both clusters, although there was evidence of sub-clustering by breeding program within the two main clusters. This clustering has also been observed in other studies using SRWW populations adapted to the Southeastern US and is hypothesized to result from the large number of linked SNPs called between lines with and without a translocation from *Triticum timopheevii* Zhuk., which harbors stem rust (*Puccinia graminis* f. sp. *tritici*) and powdery mildew (*Blumeria graminis* f. sp. *tritici*) resistance genes *Sr36* and *Pm6* (Nyquist, 1962; Benson et al., 2012; Sarinelli et al., 2019; Larkin et al., 2020). The population structure was generally low, where the first three principal components only accounted for 5.23, 3.99, and 3.42% of the total genetic variation (Figure 1). There was no noticeable differentiation between the TP18_FHB population and ADV18 and the TP19_FHB population and ADV19 (Supplementary Figures 1A,B).

### Cross Validation

**FIGURE 2:**
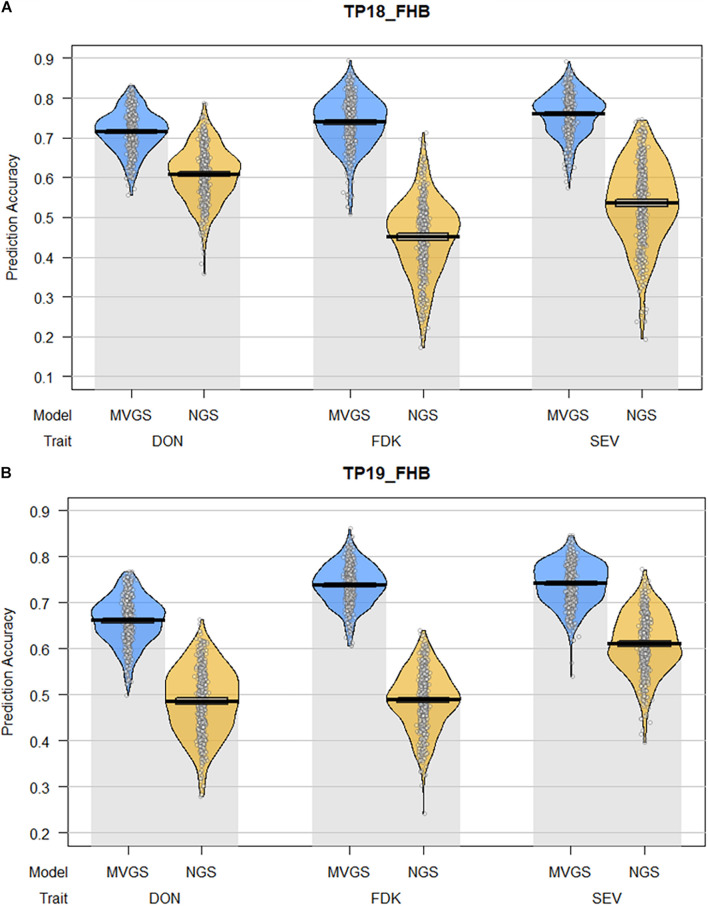
Pirate plots comparing the mean prediction accuracies between multi-trait genomic selection (MTGS) models with naïve genomic selection (NGS) models for three Fusarium head blight resistance traits (FHB), deoxynivalenol (DON) concentration, Fusarium damaged kernels (FDK), and severity (SEV) in soft red winter wheat across two training populations (TPs): **(A)** TP18_FHB, TP used to predict three FHB resistance traits for the 2018 advanced F_4:7_ generation (ADV18); **(B)** TP19_FHB, TP used to predict three FHB resistance traits for the 2019 advanced F_4:7_ generation (ADV19), consisting of all genotypes from TP18_FHB and ADV18. The *x-*axis represents the combination of FHB resistance traits and GS model used to predict each trait. The *y-*axis represents the mean prediction accuracy across 100 iterations of fivefold cross-validation in the form of a Pearson correlation coefficient (*r*) between the predicted genome-estimated breeding value (GEBV) and the actual phenotypic value for the validation populations. Individual points represent the Pearson correlation from each fold of each iteration of cross-validation for a total of 500 data points. The lines within each plot represent the mean and 95% confidence intervals for prediction accuracy. The curves represent the smoothed densities of the data.

Between both TPs, the MTGS models had significantly higher prediction accuracies compared to NGS models for DON, FDK, and SEV (Figure 2). Prediction accuracies for DON decreased between TP18_FHB and TP19_FHB while prediction accuracies for FDK and SEV increased. The decrease in prediction accuracy for DON was likely a result of background population structure within TP19_FHB between genotypes from the TP18_FHB population, which does not contain genotypes with *Fhb1*, and ADV18 which does contain genotypes with *Fhb1* (Supplementary Figure 1A). The trait with the highest mean prediction accuracies among the NGS models for TP18_FHB was DON, with a mean accuracy of 0.61, while the trait with the highest prediction accuracy for TP19_FHB was SEV (*r* = 0.61). The trait with the second highest mean prediction accuracy among the NGS models for TP18_FHB was SEV (*r* = 0.54) while DON and FDK had the same mean prediction accuracy for TP19_FHB (*r* = 0.49). Fusarium damaged kernels had the lowest mean prediction accuracy among the NGS models for TP18_FHB (*r* = 0.45). The ranking of traits between the MTGS models was not consistent with the NGS models or between TPs. Severity had the highest prediction accuracy in TP18_FHB (*r* = 0.76), followed by FDK (*r* = 0.74) and DON (*r* = 0.72). With TP19_FHB, DON also had the MTGS model with the lowest mean prediction accuracy (*r* = 0.66), while FDK and SEV had mean prediction accuracies of 0.74 (Figure 2).

### Forward Prediction

**FIGURE 3:**
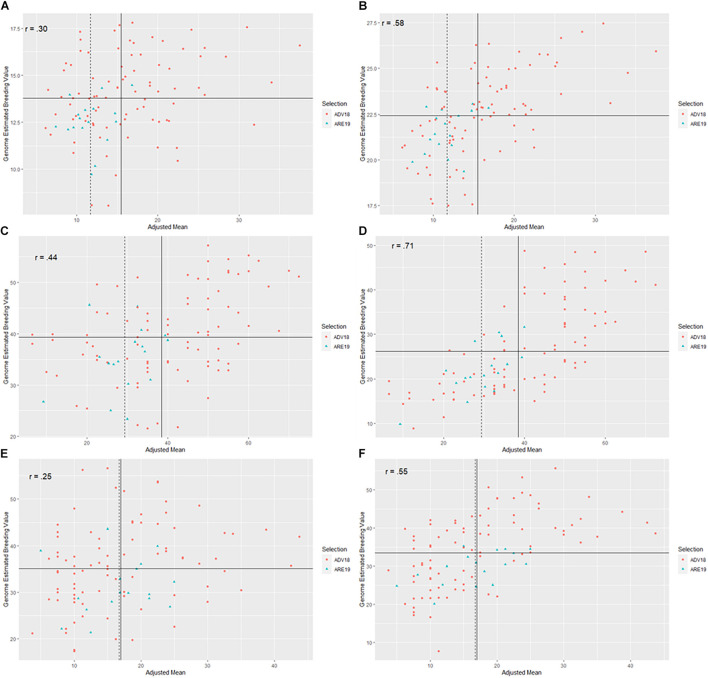
Scatter plots between genome-estimated breeding values (GEBVs) for three Fusarium head blight (FHB) resistance traits in soft red winter wheat from two different genomic selection models (GS), including naïve models without covariates (NGS) and multi-trait GS models with covariates (MTGS), and adjusted means for deoxynivalenol (DON) concentration, Fusarium damaged kernels (FDK), and severity (SEV) across two generations, F_4:7_ advanced from 2017 to 2018 (ADV18) and F_4:8_ elite from 2018 to 2019 (ARE19): **(A)** predictions for DON in ADV18 using a NGS model, **(B)** predictions for DON using a MTGS model, **(C)** predictions for FDK from ADV18 using a NGS model, **(D)** predictions for FDK using a MTGS model, **(E)** predictions for SEV in ADV18 using a NGS model, **(F)** predictions for SEV using a MTGS model. The *x*-axis represents adjusted mean for DON, FDK, or SEV across the ADV and ARE generations. The *y*-axis represents the GEBVs calculated for DON, FDK, or SEV from the NGS or MTGS models. Different colored data points represent genotypes that were advanced to the next generation. The solid vertical line represents the mean of the adjusted means for the respective FHB resistance trait from the ADV generation, while the vertical dashed line represents the mean of the adjusted means for the respective FHB resistance trait from the ARE generations. The solid horizontal line represents the mean of GEBVs for the respective FHB resistance trait calculated from the NGS or MTGS models. The *r* label represents the Pearson correlation between GEBVs and adjusted means.

When TP18_FHB was used to predict DON, FDK, and SEV for ADV18, there were significant correlations between the GEBVs calculated from the NGS and MTGS models and phenotypes for all FHB resistance traits. The strength of both correlations decreased for all methods when compared with phenotypic data from ARE19, with the exception for the MTGS model for SEV, where the correlation increased to *r* = 0.60 compared with *r* = 0.57 (Table 3). Both NGS and MTGS models had higher selection accuracies compared to phenotypic selection from ADV18 DON data (52.9%), where the NGS model correctly selected 82.4% of genotypes in ARE19, while the MTGS model correctly selected 70.6% (Table 3 and Figures 3A,B). The NGS (*R* = −0.37 μg g^–1^) model had the highest response to selection for DON compared to the NGS model (*R* = −0.23 μg g^–1^) and phenotypic selection (*R* = 0.20 μg g−^1^) (Table 3).

**TABLE 3:** Comparison of three selection methods, phenotypic selection based on three FHB resistance traits using two training populations (TP), deoxynivalenol (DON) concentration, Fusarium damaged kernels (FDK), and severity (SEV) from the advanced trials (ADV), naïve genomic selection (NGS), and multi-trait genomic selection (MTGS), based on correlations between genome estimated breeding values and the adjusted means from following generations, response to selection, and selection accuracy of genotypes in the final generation.

TP	Trait	Method	*r* ADV^a^	*r* ARE^b^	Selection differential	Response to selection	Selection accuracy
TP18_FHB	DON	PS	−	−0.01^ns†^	0.40	0.20	52.9
		NGS	0.22*	0.19^ns^	–0.73	–0.37	82.4
		MTGS	0.53***	0.10^ns^	–0.46	–0.23	70.6
	FDK	PS	−	0.14^ns^	–2.24	–1.59	58.8
		NGS	0.41***	0.38^ns^	–5.77	–4.09	70.6
		MTGS	0.70***	0.42^ns^	–3.99	–2.83	70.6
	SEV	PS	−	0.54*	–3.46	–1.49	52.9
		NGS	0.29**	0.16^ns^	–1.90	–0.82	41.2
		MTGS	0.57***	0.60*	–5.33	–2.29	47.1
TP19_FHB	DON	PS	−	0.51*	–1.32	–1.03	13.0
		NGS	0.17^ns^	0.37^ns^	–0.67	–0.53	56.5
		MTGS	0.71***	0.45*	–0.96	–0.75	69.6
	FDK	PS	−	0.67***	–4.07	–3.42	91.3
		NGS	0.18*	0.45*	–3.21	–2.70	34.8
		MTGS	0.83***	0.64**	–4.57	–3.84	60.9
	SEV	PS	−	0.78***	–5.86	–4.45	82.6
		NGS	0.25**	0.08^ns^	0.50	0.38	60.9
		MTGS	0.67***	0.12^ns^	–0.18	–0.13	82.6

*^a^Pearson correlation coefficient between GEBVs and phenotypic data from the ADV population used as a validation population (VP).*

*^b^Pearson correlations coefficient between GEBVs and adjusted means for phenotypic data from the elite (ARE) generation.*

*Significant at the 0.05 probability level.

**Significant at the 0.01 probability level.

***Significant at the 0.001 probability level.

*^†^ns, nonsignificant at the 0.05 probability level.*

When predicting FDK for ADV18, the MTGS model had the strongest correlations with the ADV18 FDK data as well as the FDK adjusted means from ARE19. The NGS (*R* = −4.09%) model again had the highest response to selection than the MTGS (*R* = −2.83%) model and phenotypic selection (*R* = −1.59%) for FDK (Table 3). The MTGS and NGS models had the same selection accuracy for FDK (70.6%) where both models outperformed phenotypic selection based on adjusted means for FDK from ADV18 (58.8%) (Table 3 and Figures 3C,D).

The MTGS model had stronger correlations between GEBVs for SEV and adjusted means for SEV from ADV18 and ARE19 than the NGS model (Table 3). The MTGS model also had the strongest response to selection (*R* = −2.29%) and selection accuracy (47.1%) compared with the NGS model, where *R* = −0.82% and selection accuracy was 41.2%. The NGS model underperformed phenotypic selection for both response to selection (*R* = −1.49%) and selection accuracy (52.9%), with the MTGS model only underperforming phenotypic selection for selection accuracy (Table 3 and Figures 3E,F).

**FIGURE 4:**
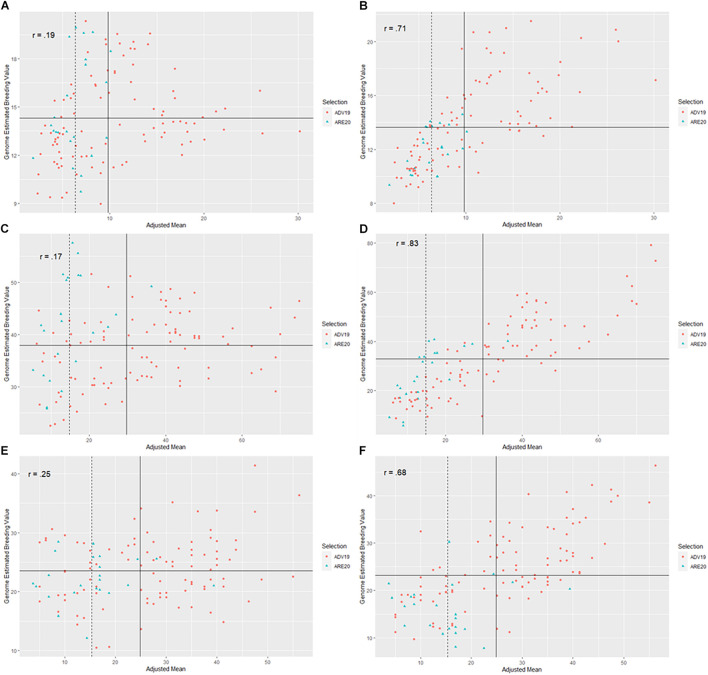
Scatter plots between genome-estimated breeding values (GEBVs) for three Fusarium head blight (FHB) resistance traits in soft red winter wheat from two different genomic selection models (GS), including naïve models without covariates (NGS) and multi-trait GS models with covariates (MTGS), and adjusted means for deoxynivalenol (DON) concentration, Fusarium damaged kernels (FDK), and severity (SEV) across two generations, F_4:7_ advanced from 2018 to 2019 (ADV19) and F_4:8_ elite from 2019 to 2020 (ARE20): **(A)** predictions for DON in ADV19 using a NGS model, **(B)** predictions for DON using a MTGS model, **(C)** predictions for FDK from ADV19 using a NGS model, **(D)** predictions for FDK using a MTGS model, **(E)** predictions for SEV in ADV18 using a NGS model, **(F)** predictions for SEV using a MTGS model. The *x*-axis represents adjusted mean for DON, FDK, or SEV across the ADV and ARE generations. The *y*-axis represents the GEBVs calculated for DON, FDK, or SEV from the NGS or MTGS models. Different colored data points represent genotypes that were advanced to the next generation. The solid vertical line represents the mean of the adjusted means for the respective FHB resistance trait from the ADV generation, while the vertical dashed line represents the mean of the adjusted means for the respective FHB resistance trait from the ARE generations. The solid horizontal line represents the mean of GEBVs for the respective FHB resistance trait calculated from the NGS or MTGS models. The *r* label represents the Pearson correlation between GEBVs and adjusted means.

When using TP19_FHB to predict FHB resistance traits for ADV19, the correlations between GEBVs from the MTGS models and phenotypic results from AVD19 were stronger than TP18_FHB for all three traits. Correlations between GEBVs from the MTGS models were stronger than TP18_FHB when compared with adjusted means from ARE20 for DON and FDK (Table 3). Response to selection for TP19_FHB was different from TP18_FHB in that phenotypic selection outperformed the GS models for DON and SEV, whereas the MTGS model had a stronger response to selection than the NGS model and phenotypic selection for FDK (Table 3). Selection accuracies did change between TPs, as the MTGS model (69.6%) outperformed both phenotypic selection (13.0%) and the NGS model (56.5%) for DON for TP19_FHB (Table 3 and Figures 4A,B). Unlike the results for TP18_FHB, both GS models had far lower selection accuracies than phenotypic selection (91.3%), although the MTGS model (60.9%) was better than the NGS model (34.8%) (Table 3 and Figures 4C,D). Selection accuracy for SEV also changed, where the MTGS model had the same selection accuracy as phenotypic selection (82.6%) while also outperforming the NGS model (60.9%) (Table 3 and Figures 4E,F).

## Discussion

Genomic selection is a valuable tool for plant breeders, and many studies have shown the vast realm of possibilities for its application (Heffner et al., 2009; Sorrells, 2015; Larkin et al., 2019). The primary goal for GS is to increase genetic gain for a trait of interest within a breeding program through the reduction of time within a breeding cycle and by improving selection accuracy (Schaeffer, 2006; Bernardo and Yu, 2007; Heffner et al., 2009; Asoro et al., 2013; Rutkoski et al., 2015). While most research in GS has focused on optimizing TPs to increase model predictive ability, less have focused on the implementation of GS into breeding programs in the form of forward selection (Bernardo, 2016). In our study, we chose to focus on forward prediction using NGS and MTGS models and compared their performance, based on selection accuracy and response to selection, to phenotypic selection for economically important traits, such as FHB resistance.

### Prediction Accuracy of Training Populations

In our study, we saw that MTGS models consistently had significantly higher prediction accuracies for DON, FDK, and SEV in every TP compared to NGS. These results were consistent with previous studies involving MTGS for FHB resistance traits (Schulthess et al., 2018; Larkin et al., 2020; Moreno-Amores et al., 2020). This follows the general trend for MTGS, where covariate traits sharing a strong correlation with a trait of interest can improve prediction accuracies for said trait of interest (Calus and Veerkamp, 2011; Jia and Jannink, 2012; Schulthess et al., 2016; Lozada and Carter, 2019; Ward et al., 2019).

Regarding the correlations between FHB resistance traits, it is interesting to note that HD was consistently negatively correlated with SEV, and yet positively correlated with DON. The negative correlation between SEV and HD has been observed in many different studies (Gervais et al., 2003; Paillard et al., 2004; Schmolke et al., 2005; Larkin et al., 2020; Moreno-Amores et al., 2020). This is because wheat genotypes that flower earlier are exposed to more favorable conditions for FHB infection, such as higher humidity and rainfall during the early growing season, versus the later part of the growing season (Buerstmayr et al., 2019). However, while positive correlations have been observed between HD and DON in other studies, less is known about this association (Liu et al., 2012; Agnes et al., 2014; Larkin et al., 2020). Agnes et al. (2014) suggested that this positive correlation was related to additional fungal growth after the soft dough stage (Feekes 11.2). Several groups have also identified QTL associated with both DON and HD (Schmolke et al., 2005; Lin et al., 2008; Agnes et al., 2014). Agnes et al. (2014) specifically identified such a QTL on chromosome 7B, which was co-located with the vernalization response gene *Vrn-B3*. Even so, like most FHB resistance traits and HD, we believe that this association is variable and environmentally dependent (Buerstmayr et al., 2019), as we saw correlations between DON and HD ranging between *r* = 0.01 and *r* = 0.31 (Table 2).

We also updated our TPs for each generation by adding phenotypic data for genotypes from the previous generation into the following year’s TP. Other studies have found that updating TPs helped to prevent the deviation in genetic relationships between the TP and VP as new germplasm was added and advanced through the breeding program (Meuwissen, 2009; Clark et al., 2012; Lorenz et al., 2012; Lorenz and Smith, 2015; Neyhart et al., 2017). Studies have also shown that larger TP sizes can have higher prediction accuracies as well, particularly when working with more diverse populations where new germplasm is continually added to the breeding program (Heffner et al., 2011; Mujibi et al., 2011; Heslot et al., 2012; Poland et al., 2012; Isidro et al., 2015; Norman et al., 2018). We also observed this trend for FDK and SEV between TP18_FHB and TP19_FHB; however, we did not observe this trend for DON, where prediction accuracy decreased when additional genotypes were added from ADV18. This can likely be attributed to less variation and a lower heritability for DON within ADV18. Genotypes within ADV18 also had the FHB resistance alleles for Fhb1, which could have increased background population structure within TP19_FHB.

### Forward Prediction

Much like the results from the cross-validation analyses of the TPs, the MTGS models had stronger correlations between their calculated GEBVs and phenotypic results from their respective VPs for FHB resistance traits, aligning with other studies involving MTGS models (Jia and Jannink, 2012; Schulthess et al., 2016; Lozada and Carter, 2019; Ward et al., 2019; Larkin et al., 2020). This was clearly observed with TP18_FHB, when correlations between MTGS GEBVs and ADV18 phenotypic results were compared with correlations between NGS GEBVs and ADV18 phenotypic results for all three traits. The prediction accuracy advantage of the MTGS model was also observed with correlations between GEBVs and ARE19 phenotypic results for FDK and SEV when compared with NGS.

Our range in prediction accuracy for the NGS models were between *r* = 0.08 and *r* = 0.45 while the range of our MTGS models was between *r* = 0.10 and *r* = 0.83. These prediction accuracies were within the range of prediction accuracies observed for FHB resistance traits in previous studies (Rutkoski et al., 2012; Arruda et al., 2015, 2016a; Larkin et al., 2020). However, the observation of lower prediction accuracies under specific circumstances was consistent with other studies with forward prediction for GY (Belamkar et al., 2018; Calvert et al., 2020). In an evaluation of forward prediction in the Kansas State University wheat breeding program, the highest prediction accuracy between the GEBVs for GY in the preliminary yield trials (PYTs) and the actual phenotypic results for GY was *r* = −0.16 (Calvert et al., 2020). The same study also used high-throughput phenotyping traits as covariates in a MTGS model for forward prediction of GY in wheat, however, the prediction accuracy was unfavorable unless a large TP was used (Calvert et al., 2020). This contrasts with our results where the use of other FHB resistance or agronomic traits as covariates significantly improved prediction accuracy for both TPs.

The MTGS model was also superior to phenotypic selection based on ADV18 phenotypic data for all three traits; however, this advantage disappeared when implementing the models trained with TP19_FHB. This is likely because genotypes in ADV19 had a much higher prevalence of resistance alleles for *Fhb1* compared with TP19_FHB, therefore the TP failed to account for this major source of genetic resistance to FHB in the VP. This highlights the importance of the TP being able to account for population structure existing within the VP, otherwise prediction accuracies can be lower. Such a result was foreshadowed with the lower prediction accuracies from the cross-validation for TP19_FHB, where no genotypes from the initial TP18_FHB contained resistance alleles for *Fhb1*, while only a small portion of genotypes from ADV18 contained the resistance alleles. A more detailed description of major and minor FHB resistance QTL present within TP18_FHB can be found in Larkin et al. (2020).

Response to selection was measured as the difference between the mean of the top 50% of breeding lines in the ARE generation, selected based on GEBVs and adjusted means of FHB resistance traits for the ADV population, compared with the mean of the full ARE population. Other studies have shown that GS could not have as high of a response to selection as phenotypic selection; however, our method of excluding phenotypic data from the ADV genotypes from the selection dataset allowed for greater independence from bias toward the phenotypic selection method (Lozada et al., 2019). In terms of response to selection, both GS models were superior to phenotypic selection for DON and FDK, and the MTGS model for SEV, when using the TP18_FHB to predict ADV18. Much like the results for prediction accuracy, this strong advantage was not observed when using the TP19_FHB to predict ADV19, except for the MTGS model for FDK, likely due to the same reasons described above. There have been no extensive forward prediction studies for FHB resistance traits in wheat. Regardless, the fact that phenotypic selection did not significantly outperform the MTGS model across years or traits indicates that MTGS models may be a good supplement, if not substitute for phenotypic selection, particularly during years when it is difficult to phenotype.

When comparing GS models with phenotypic selection for FHB resistance traits based on selection accuracy, the NGS and MTGS models had higher selection accuracies for DON using TP18_FHB, and the MTGS model was equal to phenotypic selection using TP19_FHB. Both the MTGS and NGS models were equally more accurate than phenotypic selection for FDK with TP18_FHB. Additionally, the MTGS model was equal in performance with phenotypic selection for SEV in TP19_FHB. It has been mentioned that prediction accuracy does not necessarily correlate with selection accuracy for forward prediction (Belamkar et al., 2018).

## Conclusion

This study showed that both NGS and MTGS could be successfully implemented into a SRWW breeding program, while using other agronomic and disease traits as covariates with reasonable accuracy compared to phenotypic selection and again asserted its value as a tool for plant breeders. We also found that MTGS models performed significantly better than NGS models in terms of both cross-validation within TPs as well as forward prediction of untested genotypes for economically important traits, such as FHB resistance traits. This was particularly evident when there was a strong correlation between the trait of interest and the covariate trait. This is one of the first studies to show that MTGS could be effectively implemented for forward prediction within a wheat breeding program. This is also the first study to extensively investigate the use of forward prediction when breeding for FHB resistance in wheat. We found that GS could serve as a suitable, albeit imperfect, alternative to phenotypic selection when implemented during years where environmental conditions prohibit accurate phenotypic selection, particularly when experiencing late freezing events or extensive lodging.

Prior to implementing GS into their own breeding programs, breeders must consider the genetic relationships between their prospective TPs and the breeding lines they hope to use as their VP. In the case of MTGS, breeders must also consider the correlations between their traits of interest and secondary traits used as covariates, as these correlations can differ between the TP and VP. For example, there could be a strong correlation between DON and HD in the TP but there could be a weak correlation between the two traits in the VP, therefore the MTGS model might not be more accurate than a NGS model. Inversely, there could be a strong correlation between traits in the VP while there is a weak correlation between traits in the TP, therefore MTGS could be more accurate than expected when forward prediction is implemented.

## Data Availability Statement

The data presented in the study are deposited in the FigShare repository, accession numbers https://doi.org/10.6084/m9.figshare.16685902.v1, https://doi.org/10.6084/m9.figshare.16685701.v2, https://doi.org/10.6084/m9.figshare.16685797.v1, https://doi.org/10.6084/m9.figshare.16685722.v1, and https://doi.org/10.6084/m9.figshare.16685707.v1.

## Author Contributions

DL and RM conceived and designed the experiments. DL performed data analyses, conducted the experiments, and wrote the manuscript. DM and AH collected data from the disease nurseries. RM, BW, AH, DM, and GB-G edited the manuscript. GB-G and BW conducted genotyping and generated hapmaps. All authors contributed to the article and approved the submitted version.

## Conflict of Interest

The authors declare that the research was conducted in the absence of any commercial or financial relationships that could be construed as a potential conflict of interest.

## Publisher’s Note

All claims expressed in this article are solely those of the authors and do not necessarily represent those of their affiliated organizations, or those of the publisher, the editors and the reviewers. Any product that may be evaluated in this article, or claim that may be made by its manufacturer, is not guaranteed or endorsed by the publisher.
