# Supplementary material for: Predicting Fusarium Head Blight Resistance for Advanced Trials in a Soft Red Winter Wheat Breeding Program With Genomic Selection
Source: Front Plant Sci. 2021 Oct 22;12:715314. doi: 10.3389/fpls.2021.715314 (PMC8569947; doi:10.3389/fpls.2021.715314)
Supplement: Supplementary file 1 [file Data_Sheet_1.docx]

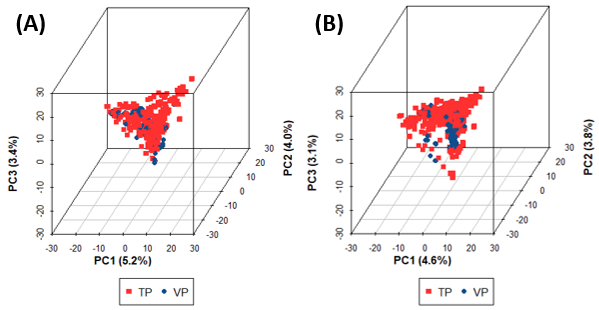


Supplementary Figure S1. Population structure between training populations (TP) used to predict FHB resistance traits for validation populations (VP) using 5,202 single nucleotide polymorphism (SNP) markers. Colors represent the population type. (A) TP, training population consisting of genotypes from TP18_FHB to predict FHB resistance traits; VP, validation population consisting of genotypes from the F_4:7_ 2018 advanced nursery (ADV18). (B) TP, training population consisting of the same genotypes from TP18_FHB and ADV18 to predict FHB resistance traits (TP19_FHB); VP, validation population consisting of genotypes from the F_4:7_ 2019 advanced nursery (ADV19). PC, principal component.
